# Rheological impact and economic implications of partial to total substitution of imported bentonite clay for oil and gas drilling operations in Nigeria

**DOI:** 10.1007/s13202-020-00963-9

**Published:** 2020-12-04

**Authors:** Oghenerume Ogolo, Akeem Arinkoola, Samuel Osisanya, Frank Egede, Ternenge Joseph Chior

**Affiliations:** 1 Department of Petroleum Engineering, African University of Science and Technology, Abuja, Nigeria; 2 Department of Chemical Engineering, Ladoke Akintola University of Technology, Ogbomosho, Nigeria; 3 Khalifa University of Science and Technology, Abu Dhabi, UAE; 4 Petroleum Training Institute, Effurun, Delta State Nigeria; 5 Petroleum and Gas Engineering Department, Nile University of Nigeria, Abuja, Nigeria

**Keywords:** Bentonite clay, Rheology, Water-based mud, Partial substitution of bentonite and economic implications

## Abstract

In less than a decade, there have been two global meltdowns of crude oil price and the latest was caused by the spread of coronavirus disease (COVID-19) in 2020. This is expected to have a negative impact on the global economy, especially on those countries that depend more on the revenue from sales of crude oil. One of the measures that can be taken to survive this kind of situation in the future is to reduce the unit technical cost for producing a barrel of oil by using locally available materials. This research investigated a local clay sourced from Ropp in Plateau State, Nigeria, by considering its rheological characteristics and economic implications of using it for partial to total substitution of imported bentonite clay for oil and gas drilling operations. The local clay was termed as Ropp bentonite clay (RBC). Various spud mud samples were prepared by dispersing a mixture of imported bentonite clay (IBC) and RBC (0–100%) in 350 ml of water. Certain quantity (0–1 g) of polyacrylamide cellulose was added to the mud samples before rheological and physical properties were determined using the standard API procedure. An economic model was built to determine the cost implications of using any of the mud formulations at different consumption rates. The results show that IBC–RBC blend in the right proportion could save Nigeria 12 to 36% of the cost of bentonite clay used to drill wells in the country.

## Introduction

The oil and gas industry has been the main stay of the Nigerian economy for many decades (Afolabi et al. 2017). It was reported that the industry accounted for about 90% of foreign earnings and 70% of government’s revenue in 2017. In less than a decade, the industry has faced two global meltdowns of crude oil price which puts the economy of the country in a chaotic condition. One was the fall in oil price in 2015 due to the breakthrough in the production of shale oil, and the other is the current and ongoing global pandemic situation caused by coronavirus disease (COVID-19) that also led to the fall in crude oil price. To minimize the effects of these challenges, it is desirable to key in to the local content initiatives of the government which encourages usage of local materials that are abound in the country so as to reduce the unit technical cost (UTC) for producing a barrel of oil in Nigeria. Also, with this initiative, the economy of the country would improve and there would be tremendous reduction in importation of foreign materials that are been used in the industry. This will also encourage indigenous company to thrive as a result of the reduction in the cost of producing a barrel of oil.

In 2010, the government of Nigeria signed the local content act into law to encourage the usage of Nigerian natural resources in the country (Afolabi et al. 2017). Nigeria is blessed with abundant natural resources, and some of them can be harnessed and used in the oil and gas industry. One of the areas of the industry where these resources can be used is drilling operations. Raw Materials Research and Development Council (RMRDC) in 2010 estimated that Nigeria consumed about 200,000 metric ton of bentonite clay for drilling operations, and most of them were imported into the country. Bentonite is used for the formulation of drilling fluid to provide the primary mud rheological property needed for drilling operations. One of the major functions of drilling fluid is to lift cuttings from the wellbore to the surface during oil and gas drilling operations (Adams 1985; Darley and George 1988).

Successful drilling operations are highly dependent on the properties and performance of drilling fluid (Darley and George 1988). This function of drilling fluid is tied to the rheological behavior of the fluid. Water-based drilling fluid is one of the most commonly used types, and it comprises a mixture of water, bentonite clay and additive (Aghamelu and Okogbue 2015; Baba Hamed and Belhadri 2009; Darley and George 1988; Jain and Mahto 2015; Mahto and Sharma 2004; Meng et al. 2012; Salawudeen et al. 2016; Wilfred and Akinade 2016).

Nigeria has a huge reserve of bentonite clay that is abound in the country, and it is reported that a substantial amount of these resources is found in every region of the country (Afolabi et al. 2017). It will be of great value to our economy and indigenous companies if these local bentonite clay resources can be used for drilling operations in the country. Though the type of bentonite found in most part of the country is calcium-based and potassium-based which has challenges of not providing the necessary rheological properties (Afolabi et al. 2017; Darley and George 1988).

Bentonite clay exists in the northeastern part of Nigeria, notably Yobe, Borno, Adamawa and Taraba states, with a probable reserve of over 700 million tonnes (Afolabi et al. 2017; Agwu et al. 2015). Also, over 90 million tonnes have been reported to have been found in some areas in Edo state, notably Ekpoma-Igunebon road, Afuze, Ovibiokhuan and Okpebho (RMRDC 2010). A probable reserve of clay of over 4 billion tons has been estimated to be found in the Niger Delta region. Oyawoye and Hirst (1964) discovered that montmorillonite clay was also present in the middle belt region of Nigeria. Their studies of younger granite at Ropp showed that a montmorillonite mineral occurred in the marginal facies of biotite–granite that are in the younger granite ring complex at Ropp, Plateau State. The deposit of bentonite clay in this location has not been investigated for use during operations.

Most of the bentonite clay deposits found in the country do not possess the rheological properties stipulated by the American Petroleum Institute (API). They have poor swelling capacity compared to the imported sodium-based bentonite clay (Agwu et al. 2015; Arabi et al 2017, 2018; Dewu et al. 2011). API stipulates that the minimum viscosity requirement for usage of any bentonite clay should be 30 cP at 600 rpm (API 1993). Most bentonite clay studied in the country falls short of this requirement. A lot of researches have been conducted on the improvement to Nigerian bentonite clay for oil and gas drilling operations. It was observed that the approach used did not improve the mud viscosity much except in cases where viscosifiers were added to the mud (Agwu et al. 2016; Apugo-Nwosu et al. 2011; Arabi et al. 2017, 2018; Arinkoola et al. 2019; Falode et al. 2007; Omole et al. 1989). Hence, the industry still relies on imported bentonite clay.

This research therefore considered the partial to total replacement of imported bentonite clay (IBC) by investigating the rheological impact and economic implication of mixing imported bentonite clay with Nigerian bentonite clay. This is aimed at providing a template to oil companies and oil and gas producing countries to partially or totally substitute imported bentonite clay with locally sourced bentonite clay during drilling operations. The study also provides the economic basis for embarking on gradual replacement of IBC with local bentonite clay.

## Theoretical framework

### Rheology

Rheological properties affect all drilling fluid functions and enable estimation of flow regimes, frictional pressure losses, equivalent circulating and surge and swab pressure which are parameters that affect hole cleaning efficiency (Guo and Liu 2011). Rheology of drilling mud is described by properties such as viscosity, yield point and gel strength.

#### Viscosity

Viscosity is a measure of the ability of fluids to resist flow. It is expressed mathematically as the ratio of shear stress to shear rate. As provided in Eq. 1, the viscosity is the ratio of shear stress to shear rate for a Newtonian fluid:

1 $$ {\text{Viscosity}}\;\mu = \frac{{{\text{Shear}}\;{\text{stress}},\tau }}{{{\text{Shear}}\;{\text{rate}}, \dot{\gamma }}} $$

Drilling fluids are non-Newtonian, and hence, there is no single viscosity value at various shear rates. Non-Newtonian fluids are either shear rate or shear-time dependent. Non-Newtonian fluids are grouped into pseudo-plastics and dilatant. Pseudo-plastic fluid exhibits decreasing apparent viscosity with increasing shear rates, and dilatants have their apparent viscosity increasing with increasing shear rate. Shear-time-dependent non-Newtonian fluids are rheopectic if the apparent viscosity of the fluid increases with time after shear rate is increased to a new constant value. Drilling fluids are described as thixotropic if the apparent viscosity decreases when shear rate is increased to a new value that is constant. Generally, drilling fluids are pseudo-plastic and thixotropic.

#### Yield point and gel strength

Drilling fluids will only flow when subjected to certain load stress called yield point (Maxey 2007). Yield point is the stress required to start fluid movement. It is caused by electrical charges located close to the surface of the particles in the mud (Sadek et al. 2011). Sami (2016) stated that gel strength is a function of interparticle forces of drilling mud and the shear stress measured at low shear rate. Gel strength and yield stress both are a measure of the same interparticle forces of a fluid as determined by the yield value. However, unlike yield point, gel strength is measured under static condition.

#### Plastic viscosity and apparent viscosity (AV)

Plastic viscosity is a measure of the internal resistance to fluid flow due to the amount, type and size of solids present in the fluid. Accumulations of drilled solids, additions of barite and presence of chemical contaminants in drilling mud increase the plastic viscosity. The dilution of drilling mud with water, effective use of solids control equipment and flow line flocculation decrease the plastic viscosity (Sadek et al. 2011). The plastic viscosity is calculated as the difference between the 600 rpm dial reading and the 300 rpm dial reading of the viscometer. The apparent viscosity is also a rheological parameter, and it is calculated by dividing the dial reading of the viscometer at 600 rpm by 2 (Bourgoyne et al. 1991). The apparent viscosity gives the measure of that part of resistance to flow caused by mechanical friction between solids in the mud, solids and liquids and the shearing layers of the mud itself.

## Methodology

### Materials and equipment

The following materials and apparatus were used to achieve the objectives of this research work: imported bentonite clay, local bentonite clay, distilled water, spatula and measuring cylinders. While the equipment used includes: variable mud mixer (Ofite, Model 9B), viscometer (Ofite, Model 800), pH meter (OAKTON, Model pH Tester 10), oven (U-Test GENO, DT104A), jaw crusher (Retsch, BB 50), weigh balance (OHAUS, Model AX124) and mud balance (Ofite).

### Processing of clay samples

The local bentonite clay used for this research was collected from Ropp, Plateau State, Nigeria. The raw clay sample was dried in the oven at a temperature of 70 °C for 12 h. The dried clay sample was crushed using a jaw crusher and sieved with 200 API mesh size to obtain fine particles of the clay (API 2010; Magzoub et al. 2017).

### Drilling fluid preparation and characterization

One laboratory barrel of water-based mud was prepared by mixing 22 g of RBC with 350 ml of distilled water. Six other samples of one laboratory barrel of water-based mud were prepared by varying the percentage of RBC in the clay composite as shown in Table 1. The drilling mud samples prepared were characterized using API standard testing procedures for mud property determination (API 2010). The properties of the mud determined include mud rheological properties and mud pH. It was observed that after the samples were prepared, the viscosity of all the mud samples was not up to API standard. Hence, all the mud samples were beneficiated using polyacrylamide cellulose (PAC). It was seen that with addition of 0.5 g of PAC, most of the mud samples met API standard in terms of drilling mud viscosity.

**Table 1:** Blend of clay samples

Sample	Blend	Concentration of clay (grams per laboratory barrel)	
		IBC	RBC
A	0%IBC/100%RBC	0	22
B	20%IBC/80%RBC	4.4	17.6
C	40%IBC/60%RBC	8.8	13.2
D	50%IBC/50%RBC	11	11
E	60%IBC/40%RBC	13.2	8.8
F	80%IBC/20%RBC	17.6	4.4
G	100%IBC/0%RBC	22	0

### Economics of partial to total substitution of IBC with RBC

The economic assumptions made for the cost of materials used for the drilling fluid formulation considered are shown in Table 2. The costs of one gram of RBC, IBC and PAC were $0.00042, $0.00071 and $0.0044, respectively. The costs of one ton of RBC and IBC and PAC were $250, $710 and $4400. Table 3 shows the volumetric specification for the amount of drilling fluid used for modeling the economics of the clay blend per field barrel and well basis. The cost of using any of the beneficiated clay samples (drilling mud plus PAC) for drilling operations was determined using the economic assumptions stated in Table 2. This was done on a one laboratory barrel, field barrel and well basis to see the amount of money saved for using any of mud samples prepared as a replacement of IBC.

**Table 2:** Cost of imported bentonite, local bentonite and PAC

Quantity	Cost RBC ($)	Cost of imported bentonite clay ($)	Cost of PAC ($)
1 g	0.00025	0.00071	0.00440
22 g	0.00550	0.01562	0.09680
1 sack	250.0	710.0	110.0

**Table 3:** Volumetric specification of drilling fluid

Specification	Quantity	Unit
One field barrel	159	Liters
Number of bentonite sacks per well	15	Ton

The data used for estimating the cost of IBC and amount of bentonite clay used to drill a well were obtained from a drilling program used to drill a well in Agbatu field in the onshore Niger Delta region of Nigeria. The well depth was 5900 ft TVD, and the cost of drilling the well was about $10 million. The cost of bentonite clay used to drill the well was $10,650, and it accounted for about 0.11% of the entire cost of the well. The cost of any of the clay blends on a ton basis was also determined. This was used to estimate the cost of using any of the clay blends based on a Nation's consumption capacity.

## Discussion of results

### Effect of substituting IBC with RBC on the rheological parameters of the mud

**Fig. 1:**
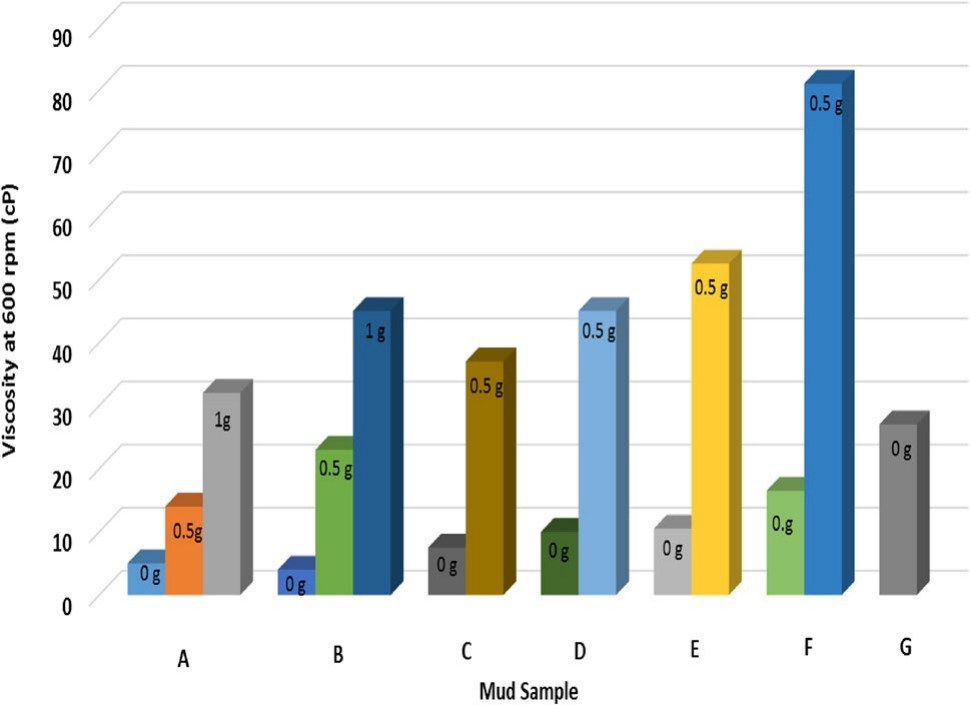
Effect of addition of PAC on the viscosity of the mud samples formulated at 600 rpm

Figure 1 shows the effect of substituting IBC with RBC on the mud viscosity at 600 rpm. API stipulates a minimum viscosity requirement for any clay that will be used for drilling operations. It states that the clay must be able to yield a viscosity of 30 cP at 600 rpm. Initially, the viscosity of RBC was 5 cP but upon blending it with IBC, it increased to 7.5, 10, 10.5, 16.5 cP when 40%, 50%, 60% and 80% of IBC were used, respectively. The maximum increment in the viscosity values was about 230%, which was when 80% of IBC was used. But still the value of the viscosity obtained was not up to API standard, and it was also less than the viscosity of sample G (27 cP) which was prepared with 100%IBC. When the samples were beneficiated using PAC, the viscosity of the mud increased beyond API standard. Though for sample A and sample B, it was increased beyond API standard when 1 g of PAC was added to it. The viscosity values obtained for sample A and sample B were 32 and 45 cP, while for the other mud samples, upon adding 0.5 g of PAC, the viscosity of the mud increased beyond API standard. The viscosity values for sample C to sample F increased to 37, 45, 52.5 and 81 cP, respectively. It is seen that blending RBC with IBC had impact on the mud viscosity but could not improve it to API standard. But addition of PAC to the mud samples was able to raise the mud viscosity to API standard.

Table 4 shows the impact of blending RBC with IBC on the apparent viscosity, plastic viscosity and yield point of the mud samples formulated, while Table 5 shows the impact of blending RBC with IBC on the gel strength, consistency factor and consistency index of the mud samples. It is seen that blending RBC with IBC improved the rheological parameters of the mud. The 10-s and 10-min gel strength increased from 0.5 to 9.5 1b/100 ft^2^ and 0.5 to 15 1b/100 ft^2^, respectively, when blends of 80%IBC and 20%RBC were used. Also, the apparent viscosity, plastic viscosity and yield point also increased with increment in the percentage of IBC. This increment in the rheological properties was as a result of blending RBC with IBC. IBC is a sodium-based bentonite clay with good swelling capacity. Hence, blending the RBC with IBC led to an improvement in the rheological parameters. The consistency factor was also increased from 0.03 to 1.52. The consistency index which indicates the flow behavior of the mud shows that the mud still behaves like a non-Newtonian fluid.

**Table 4:** Apparent viscosity, plastic viscosity and yield point of the mud samples formulated

Sample	Mass of PAC added (g)	Apparent viscosity (cP)	Plastic viscosity (cP)	Yield point (1b/100 ft^2^)
A	0.00	2.50	2.00	1.00
	0.50	7.00	4.50	5.00
	1.00	16.00	9.00	14.00
B	0.00	2.00	1.00	2.00
	0.50	11.50	7.50	8.00
	1.00	22.50	13.00	19.00
C	0.00	3.75	2.50	2.50
	0.50	18.50	10.00	17.00
D	0.00	5.00	3.50	3.00
	0.50	22.50	11.00	23.00
E	0.00	5.25	3.50	3.50
	0.50	26.25	11.50	29.50
F	0.00	8.25	3.50	9.50
	0.50	40.50	15.50	50.00
G	0.00	11.50	3.00	17.00

**Table 5:** Gel strength, consistency factor and consistency index of the mud samples formulated

Sample	Mass of PAC added (g)	Gel strength		Consistency factor, *K*	Consistency index, *n*
		10 s	10 m		
		(1b/100 ft^2^)			
A	0.00	0.50	0.50	0.03	0.74
	0.50	1.00	1.00	0.29	0.56
	1.00	2.50	30.00	1.18	0.48
B	0.00	0.50	0.50	0.23	0.41
	0.50	2.00	6.00	0.45	0.57
	1.00	5.00	12.00	1.49	0.49
C	0.00	1.00	3.00	0.13	0.58
	0.50	7.00	26.00	1.59	0.45
D	0.00	2.00	5.00	0.14	0.62
	0.50	15.00	47.00	2.73	0.40
E	0.00	3.00	7.00	0.18	0.58
	0.50	23.00	63.00	4.44	0.36
F	0.00	9.50	15.00	1.52	0.34
	0.50	40.00	93.00	9.70	0.31
G	0.00	19.00	24.00	5.69	0.20

Addition of PAC to the mud samples shows a great improvement in the rheological parameters. PAC was added to the mud samples to ensure that the mud rheological parameters meet up with API standard. Addition of 0.5 g of PAC increased the 10-s gel strength for sample A from 0.5 to 1.0 1b/100 ft^2^ and from 3 to 40 1b/100 ft^2^ for sample F. The yield point increased from 1.0 to 5.0 1b/100 ft^2^ for sample A and from 9.5 to 50.0 1b/100 ft^2^ for sample F. The increment in the yield point is beyond API standard as API requires a maximum of 30 1b/100 ft^2^ for the yield point. But for sample E, addition of 0.5 g of PAC to the mud sample increased the yield point of the mud from 3.5 to 29.5 1b/100 ft^2^, which is in the range of API standard. The plastic viscosity of the mud at this point was increased from 3 to 11.5 cP. It was increased beyond the maximum value set by API (8–10 cP). Samples A with addition of 1 g of PAC increased the plastic viscosity from 2.0 to 9.0 cP, while sample C with addition of 0.5 g of PAC increased the plastic viscosity from 2.5 cP to 10 cP which is in the range of API standard.

### Effect of substituting IBC with RBC on the shear stress/shear rate relationship of the mud

**Fig. 2:**
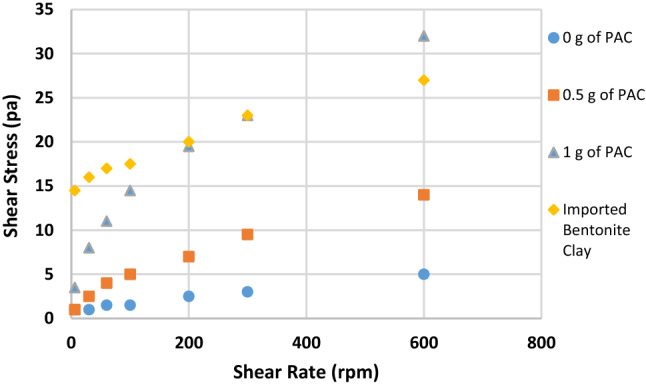
Shear stress/shear rate relationship of mud (sample A)

**Fig. 3:**
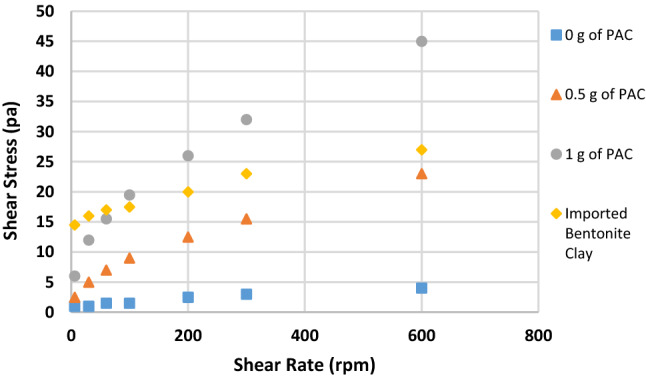
Shear stress/shear rate relationship of mud (sample B)

**Fig. 4:**
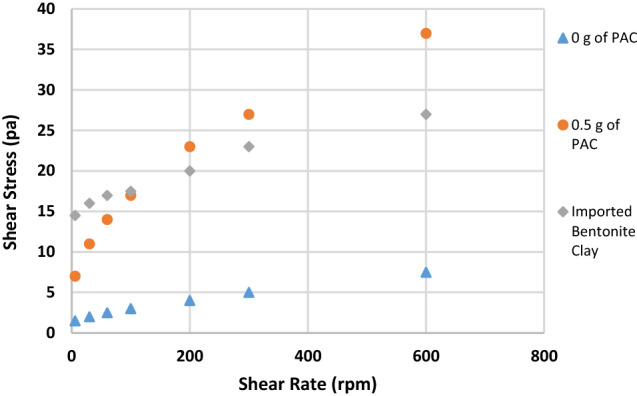
Shear stress/shear rate relationship of mud (sample C)

**Fig. 5:**
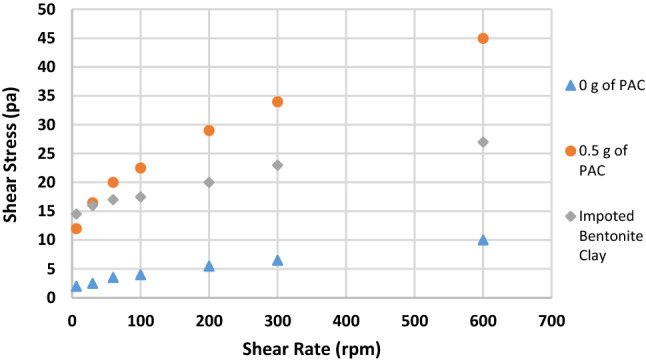
Shear stress/shear rate relationship of mud (sample D)

**Fig. 6:**
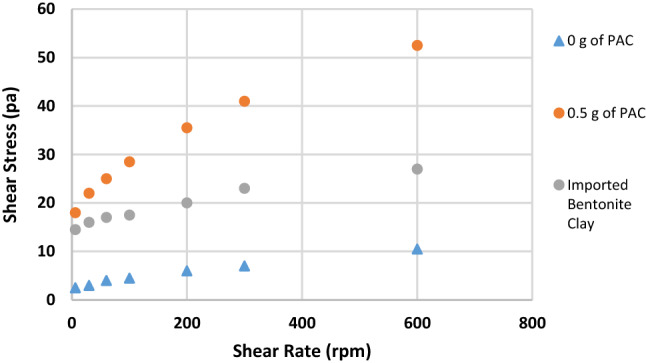
Shear stress/shear rate relationship of mud (sample E)

**Fig. 7:**
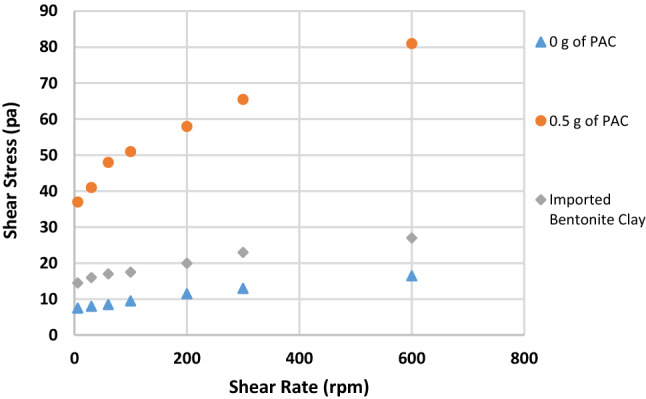
Shear stress/shear rate relationship of mud (sample F)

**Fig. 8:**
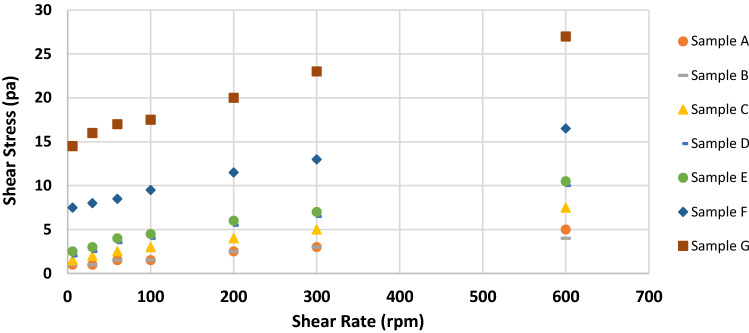
Shear stress/shear rate relationship of all the mud samples without PAC

Figures 2, 3, 4, 5, 6 and 7 show the impact of the substitution process on the shear stress/shear rate relationship of the various mud samples considered. The mud samples prepared with 100%RBC had a very low shear stress relationship indicating a poor viscous nature of the fluid as seen in Fig. 2. But upon blending RBC with IBC using the blending ratio in Table 1, it is seen that the shear stress behavior of the mud reduced initially for sample B when 80%RBC was blended with 20%IBC and increased as the percentage of IBC increases. This is shown in Fig. 8. Figure 8 shows the shear stress/shear rate relationship of all the mud samples prepared without the addition of PAC. Blending RBC with IBC was able to increase the shear stress/shear rate relationship making the fluid to behave like a Bingham plastic fluid. Also beneficiating the mud samples prepared with PAC was able to improve the shear stress/shear rate relationship greatly.

### Effect of substituting IBC with RBC on the pH of the mud

Blending RBC with IBC had a slight impact on the mud pH. The pH of the mud prepared with 100%RBC (sample A) was 7.0, while that prepared with 100%IBC (sample G) was 8.0. The pH of sample F was 7.5 which was as a result of the high percentage of IBC present in the mud. It was observed that addition of PAC did not alter the mud pH (Table 6).

**Table 6:** Mud pH of all the mud samples formulated

Sample	Mass of PAC added (g)	pH
A	0.00	7.00
	0.50	7.00
	1.00	7.00
B	0.00	7.00
	0.50	7.00
	1.00	7.00
C	0.00	7.00
	0.50	7.00
D	0.00	7.00
	0.50	7.00
E	0.00	7.00
	0.50	7.00
F	0.00	7.50
	0.50	7.50
G	0.00	8.00

### Economics of partial to total substitution of IBC with RBC

The economic analysis of partial to total substitution of IBC with RBC at different consumption rates is discussed in this section. Table 7 shows the cost of each blend of clay with and without PAC per laboratory barrel. The costs of sample A, sample D, sample F and sample G without PAC per laboratory barrel were $0.0055, $0.0106, $0.0136 and $0.0156, respectively. Addition of PAC to the mud samples increased the cost of one laboratory barrel of each sample by $0.0022 and $0.0044 when 0.5 g and 1.0 g of PAC were added to the mud samples. The cost of sample F with 0.5 g of PAC per laboratory barrel was higher than the cost of sample G which is 100%IBC. This shows that addition of PAC for sample F increases the cost of the clay blend beyond the cost when 100% of IBC was used. Table 8 shows the cost of each blend of clay per field barrel. Similar observation was made as addition of PAC to the mud samples also increased the cost of the clay blend. As the percentage of IBC increases, the cost of the clay blend also increases. This is because IBC is more expensive than RBC. Hence, the higher the percentage of IBC in the mud sample, the higher the cost of the clay blend. On a field barrel basis, the costs of sample A, sample D, sample F and sample G without PAC were $2.50, $4.80, $6.12 and $7.10.

**Table 7:** Cost of blend of clay with PAC per laboratory barrel of mud

Clay blend	Cost of blend of clay per laboratory barrel ($)		
	0 g of PAC	0.5 g of PAC	1 g of PAC
A	0.0055	0.0077	0.0099
B	0.0075	0.0097	0.0119
C	0.0095	0.0117	0.0139
D	0.0106	0.0128	0.0150
E	0.0116	0.0138	0.0160
F	0.0136	0.0158	0.0180
G	0.0156	–	–

**Table 8:** Cost of blend of clay with PAC per field barrel of mud

Clay blend	Cost of blend of clay per field barrel ($)		
	0 g of PAC	0.5 g of PAC	1 g of PAC
A	2.50	3.50	4.50
B	3.42	4.42	5.42
C	4.34	5.34	6.34
D	4.80	5.80	6.80
E	5.26	6.26	7.26
F	6.18	7.18	8.18
G	7.10	–	–

**Fig. 9:**
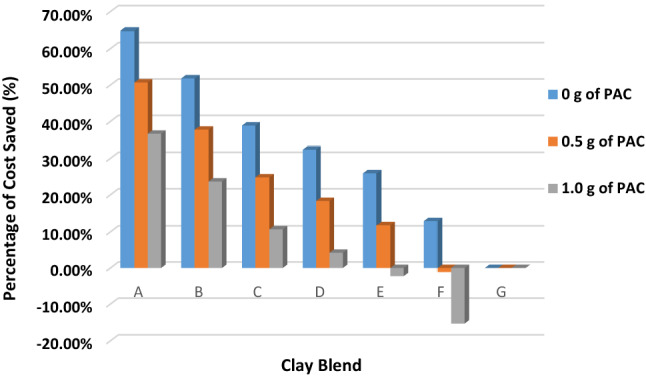
Percentage of cost saved per clay blend sample selected

The economic impact of partial to total substitution of IBC with RBC on a well basis is shown in Table 9. It is seen that the costs of using sample A, sample D, sample F without PAC to drill the well were $3750, $7200 and $9270 and with 0.5 g of PAC were $5250, $8700 and $10,770. The cost of imported bentonite clay used to drill the well in Agbatu field was $10,650. It is observed also that the cost of sample F with 0.5 g of PAC was higher than the cost of sample G, which means more money will be spent for usage of such clay blend which is not desirable. But for usage of sample A to sample E with PAC, a company can save about 12% to 36% of the cost of bentonite clay used to drill a well. This can be seen in Fig. 9. Figure 9 shows the percentage of cost saved per usage of any of the blends of clay. It is also seen that blending RBC with IBC alone could make a company save about 13% to 51% of the cost of bentonite. The blend of clay without PAC could be used to drill shallow sections of the well, and as the depth increases, the use of PAC becomes inevitable in most cases.

**Table 9:** Cost of blend of clay with PAC per well drilled

Clay blend	Cost of blend of clay per well ($)		
	0 g of PAC	0.5 g of PAC	1 g of PAC
A	3750	5250	6750
B	5130	6630	8130
C	6510	8010	9510
D	7200	8700	10,200
E	7890	9390	10,890
F	9270	10,770	12,270
G	10,650	–	–

Table 10 shows the cost of using any of the clay blends with and without PAC on a ton basis. The cost of a ton of sample A (100%RBC) was $250 and sample G (100%IBC) was $710. The costs of a ton of sample C, sample E and sample F without PAC were $434, $526 and $618, while with 0.5 g of PAC, the costs were $534, $626 and $718. The cost of sample F with PAC is undesirable as it is more expensive than sample G (100%IBC). The cost of using any of the clay blends as shown in Table 10 can be used to determine the cost implication of substituting IBC with RBC based on a Nation's consumption capacity. In 2010, RMRDC estimated that Nigeria consumed 200,000 metric tonnes of bentonite clay. Using any of the acceptable clay blends could have saved the country about $17,040,000 to $51,120,000.

**Table 10:** Cost of blend of clay with PAC based on a Nation’s consumption capacity

Clay blend	0 g of PAC ($M)	0.5 g of PAC ($M)	1 g of PAC ($M)
*1 ton*			
A	250	350	450
B	342	442	542
C	434	534	634
D	480	580	680
E	526	626	726
F	618	718	818
G	710	–	–
*1,000 ton*			
A	250	350	450
B	342	442	542
C	434	534	634
D	480	580	680
E	526	626	726
F	618	718	818
G	710	–	–
*10,000 ton*			
A	2.5	3.5	4.5
B	3.42	4.42	5.42
C	4.34	5.34	6.34
D	4.8	5.8	6.8
E	5.26	6.26	7.26
F	6.18	7.18	8.18
G	7.1	–	–
*20,000 ton*			
A	5	7	9
B	6.84	8.84	10.84
C	8.68	10.68	12.68
D	9.6	11.6	13.6
E	10.52	12.52	14.52
F	12.36	14.36	16.36
G	14.2	–	–
*30,000 ton*			
A	7.5	10.5	13.5
B	10.26	13.26	16.26
C	13.02	16.02	19.02
D	14.4	17.4	20.4
E	15.78	18.78	21.78
F	18.54	21.54	24.54
G	21.3	–	–
*40,000 ton*			
A	10	14	18
B	13.68	17.68	21.68
C	17.36	21.36	25.36
D	19.2	23.2	27.2
E	21.04	25.04	29.04
F	24.72	28.72	32.72
G	28.4	–	–
*50,000 ton*			
A	12.5	17.5	22.5
B	17.1	22.1	27.1
C	21.7	26.7	31.7
D	24	29	34
E	26.3	31.3	36.3
F	30.9	35.9	40.9
G	35.5	–	–
*60,000 ton*			
A	15	21	27
B	20.52	26.52	32.52
C	26.04	32.04	38.04
D	28.8	34.8	40.8
E	31.56	37.56	43.56
F	37.08	43.08	49.08
G	42.6	–	–
*70,000 ton*			
A	17.5	24.5	31.5
B	23.94	30.94	37.94
C	30.38	37.38	44.38
D	33.6	40.6	47.6
E	36.82	43.82	50.82
F	43.26	50.26	57.26
G	49.7	–	–
*80,000 ton*			
A	20	28	36
B	27.36	35.36	43.36
C	34.72	42.72	50.72
D	38.4	46.4	54.4
E	42.08	50.08	58.08
F	49.44	57.44	65.44
G	56.8	–	–
*90,000 ton*			
A	22.5	31.5	40.5
B	30.78	39.78	48.78
C	39.06	48.06	57.06
D	43.2	52.2	61.2
E	47.34	56.34	65.34
F	55.62	64.62	73.62
G	63.9	–	–
*100,000 ton*			
A	25	35	45
B	34.2	44.2	54.2
C	43.4	53.4	63.4
D	48	58	68
E	52.6	62.6	72.6
F	61.8	71.8	81.8
G	71	–	–
*150,000 ton*			
A	37.5	52.5	67.5
B	51.3	66.3	81.3
C	65.1	80.1	95.1
D	72	87	102
E	78.9	93.9	108.9
F	92.7	107.7	122.7
G	106.5	–	–
*200,000 ton*			
A	50	70	90
B	68.4	88.4	108.4
C	86.8	106.8	126.8
D	96	116	136
E	105.2	125.2	145.2
F	123.6	143.6	163.6
G	142	–	–

## Conclusions

Blending RBC with IBC had an impact on the rheological behavior of the mud. The viscosity at 600 rpm of the mud prepared with 100%RBC was 5 cP, and it was improved to 17.5 cP. The viscosity values for all the blends of clay obtained were not up to API standard. But upon blending it with PAC, the viscosity of the mud met with API standard.

Also, addition of PAC to the different mud samples increased other rheological parameters of the mud making some of the blends of clay suitable for drilling operations as they were able to meet up with API standard.

The economic analysis reveals that an oil company or oil and gas nation could save 12 to 36% of the cost of bentonite clay used for drilling operations at different rates of consumption. Also, it was seen that using sample F which consists of 80%IBC and 20%RBC with PAC is undesirable as the cost of the sample exceeds the cost of using 100% imported bentonite clay (sample G).

## Abbreviations

IBC: Imported bentonite clay
PAC: Polyacrylamide cellulose
RBC: Ropp bentonite clay
RMRDC: Raw Materials Research and Development Council
UTC: Unit technical cost
